# The Heterogeneity of Asthma Phenotypes in Children and Young Adults

**DOI:** 10.1155/2012/163089

**Published:** 2012-04-11

**Authors:** Bill Hesselmar, Anna-Carin Enelund, Bo Eriksson, Leonid Padyukov, Lars Å. Hanson, Nils Åberg

**Affiliations:** ^1^Department of Pediatrics, Institute of Clinical Sciences, Sahlgrenska Academy, 41685 Gothenburg, Sweden; ^2^Queen Silvia Children's Hospital, 41685 Gothenburg, Sweden; ^3^Nordic School of Public Health, 40242 Gothenburg, Sweden; ^4^Rheumatology Unit, Department of Medicine, Karolinska Institutet, 17176 Stockholm, Sweden; ^5^Department of Microbiology and Immunology, Institute of Biomedicine, Sahlgrenska Academy, Gothenburg, Sweden

## Abstract

*Objective*. Genetic heterogeneity and risk factor distribution was analyzed in two previously proposed asthma phenotypes. *Method*. A sample of 412 subjects was investigated at 7-8, 12-13, and 21-22 years of age with questionnaires, skin prick tests, and genetic analysis of IL-4 receptor (IL4R) single-nucleotide polymorphisms. The sample was subdivided in one group with no asthma, and two groups with asthma separated by age of onset of symptoms, namely, early onset asthma (EOA) and late onset asthma (LOA). Risk factors and IL4R markers were analyzed in respect to asthma phenotypes. *Results*. EOA and LOA groups were both associated with atopy and a maternal history of asthma. Female gender was more common in LOA, whereas childhood eczema, frequent colds in infancy, and a paternal history of asthma were more common in EOA. The AA genotype of rs2057768 and the GG genotype of rs1805010 were more common in LOA, whereas the GG genotype of rs2107356 was less common in EOA. *Conclusion*. Our data suggest that early and late onset asthma may be of different endotypes and genotypes.

## 1. Introduction

Asthma is a common disease, but it has been questioned if it is one single disease, or a group of asthmatic diseases. Such different “phenotypes” of asthma may vary in response to treatment [1], in prognosis [2], in inflammatory patterns [3], and in susceptibility to environmental exposure [4]. Identifying possible subphenotypes has therefore rendered increasing interest in recent years [5, 6]. But a reliable subgroup classification of asthma (or any other disease) can seldom include only clinical parameters, very often additional biomarkers have to be included [7] in order to find genetic or endotypic differences.

In the 1990s, the Tucson group presented data on lung function of infants from a population-based birth-cohort study and they also retrospectively classified preschool wheeze into three subgroups or phenotypes, namely, transient wheeze, early persistent wheeze, and late onset asthma [8]. Interestingly, not only transient wheeze had a characteristic phenotype pattern, there were also differences between the two asthma subgroups, with eczema being more common among children with early persistent wheeze than among children with asthma onset after the age of 3 years. If the noticed difference in the prevalence of eczema in children with early versus late onset of respiratory symptoms is of any phenotypic significance is, however, not known. Our own data reveal that 12-13-year-old children with asthma and eczema differ regarding the interleukin-4 receptor genotype from children with asthma, but with no eczema [9]. On the other hand, we have also seen that the same genetic pattern in children may have different consequences in adult women with severe atopic asthma [10]. If these contrasting results are due to different asthma phenotypes being tested, or to random associations in different studies, is still not known. However, since the association between asthma and eczema resembles the phenotype “early persistent wheeze” used in the Tucson study, and there is a predominance of females among adults with asthma, we wanted to investigate if early onset asthma (<3 years) has phenotypic and genotypic differences as compared to asthma starting at an older age.

The aim of the present study was to apply, as close as possible, the Tucson-based phenotypes “early persistent wheeze” and “late onset asthma” on a cohort of children, followed from 7-8 to 21-22 years of age, in order to test if these “phenotypes” have different patterns in inherent risk factors for asthma, in asthma-associated features, and in genetic markers from the IL-4-receptor.

## 2. Methods

### 2.1. Subjects

**Figure 1:**
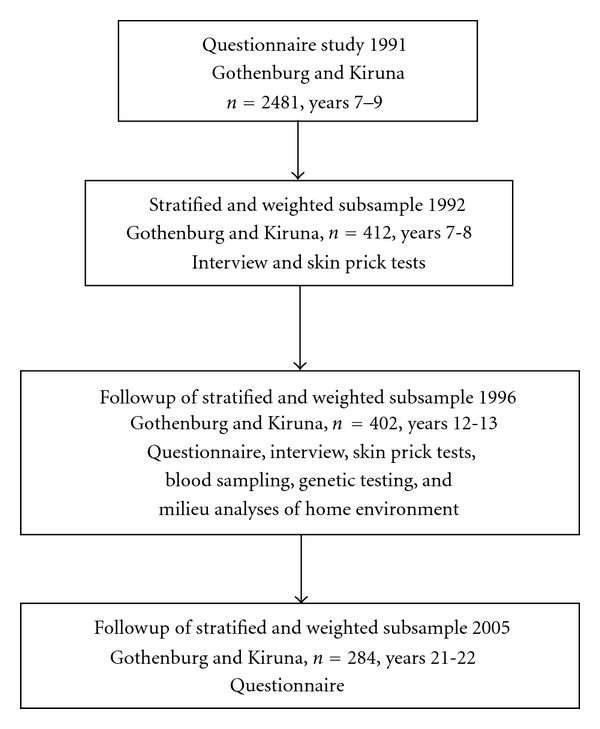
Flow-chart of study design and followup.

A cross-sectional questionnaire study was performed in 1991, covering all 7–9-year-old children in Kiruna, a town in the northernmost part of Sweden, and a selected sample of 7-year-old children in Gothenburg, a city on the south-west coast of Sweden. In Gothenburg we choose a sample of schools covering known variations in socioeconomy and in environmental pollution. The cohort comprised 2481 children. A stratified subsample of 412 7-8-year-old children were chosen for further interviews and allergy testing in 1992. In 1996, when the children had reached 12-13 years of age, they were once again contacted for a followup. 402 children accepted to take part in the followup, which included a questionnaire, an interview, allergy testing, and blood sampling. The recruiting procedure, the power calculations, and the stratification procedure have previously been described in detail [11, 12]. In 2005, a second questionnaire followup was performed. Two hundred and eighty-four subjects, with an age of 21-22 years, agreed to participate; 152 women and 132 men. Of those who in 2005 accepted to participate, 35 (12.3%) reported current asthma at 12-13 years of age. The corresponding figure for the 118 missing subjects was 15 (12.7%), (*P* = 0.915, Chi-square). A flow chart of the recruiting and follow-up procedure is presented in Figure 1.

The questions about current and former disease in the followup were similar as in the original cross-sectional study from 1991 [11]; as to asthma and eczema we asked: have you/your child ever had asthma or asthmatic bronchitis? Have you/your child ever had eczema? Have there been any symptoms in the last year?

From retrospective questionnaire data at 7 years of age, we collected information on symptoms and disease during the preschool years, as well as information on age when symptoms of disease were first noted, the number of colds the child had during the first year of life, and if there was a parental history of asthma. Data on current asthma and eczema, that is symptoms within the last 12 months, were collected at 7-8 years, 12-13 years, and 21-22 years of age.

### 2.2. Allergy Testing

Allergy testing with skin prick tests (SPT) was done at 7-8 years of age. The test was carried out according to the standards of the subcommittee on skin tests of the European Academy of Allergy and Clinical Immunology [13]. The allergen extracts used were all of ALK make (ALK, Hørsholm, Denmark); Soluprick SQ 10 HEP: birch, timothy, dog, cat, horse, *Dermatophagoides pteronyssinus,* and *Dermatophagoides farinae*. *Cladosporium herbarum* was represented by Soluprick in dilution 1 : 20. Positive control was histamine 10 mg/mL Soluprick. A positive SPT corresponds to a weal with a diameter exceeding the negative control (NaCl 9 mg/mL) by at least 3 mm. A subject was considered atopic if he/she had at least one positive skin prick test.

### 2.3. Phenotyping

We used the combined results from the original cross-sectional study in 1991, and the two follow-up studies in 1996 and 2005, to identify asthma phenotypes. 359 subjects could be classified into one healthy group and two different asthma phenotypes (Table 1).

### 2.4. Genotyping

**Figure 2:**
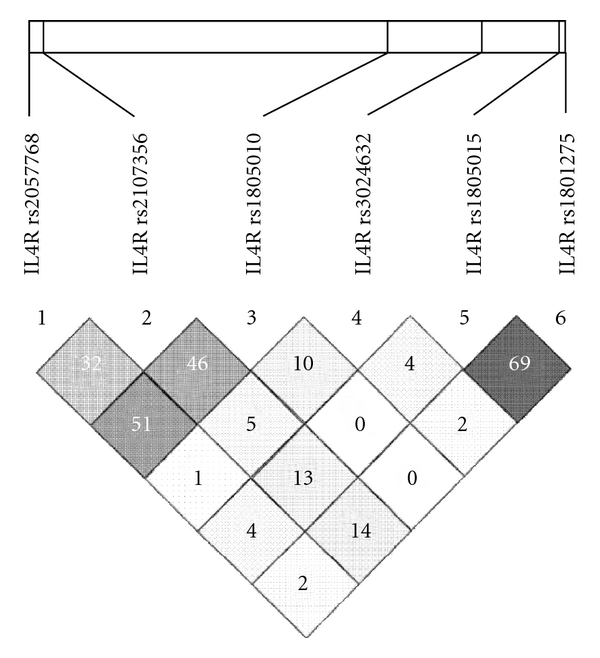
Linkage disequilibrium structure in the IL4R locus in the study population. Numbers correspond to *r*-square values calculated in Haploview.

The selection of markers and the genotyping procedure, for single-nucleotide polymorphisms (SNPs) in the IL4R (interleukine-4 receptor) gene has previously been described by us [9, 10, 14]. In short, plasma and buffy coat were separated from EDTA-treated venous blood samples. The buffy coats were stored at −70°C until DNA extraction. The DNA samples for genotyping were extracted from buffy coats, using the salting out method. The selected markers (rs2057768, rs2107356, rs3024632, rs1805010, rs1805015, and rs1801275) represent different linkage disequilibrium blocks along the IL4R gene (Figure 2), and they were all tested for possible associations with specific asthma phenotypes in our study as defined in Table 1. 

### 2.5. Statistics

Statistical analyses were performed with the SPSS ver. 15.0.1.1 software (SPSS, Chicago, IL, USA). Differences between groups were analysed with exact tests, and a multiple logistic regression model was used in the analysis of a potential relationship between early onset asthma and paternal asthma in order to adjust for a history of eczema in preschool years. The use of this model could be questioned since it includes two independent variables, despite the fact that there are only 11 cases with early onset asthma. We have, however, decided to present the results from the analysis since the Hosmer and Lemeshow Test showed an acceptable Goodness of Fit (*P* = 0.640).

Cross-tabulations using Monte Carlo simulation tests with 100,000 iterations were used to compare proportions between genotype and asthma phenotypes. The significance level was set to 5%.

### 2.6. Ethics

The study was approved by the Human Research Ethics Committee of the Medical Faculty, University of Gothenburg, Sweden.

## 3. Results

With the combined results from the followup at 12-13 and at 20-21 years of age, 359 of the original 412 children could be classified into early onset and late onset asthma phenotypes, as defined in Table 1. Three inherent risk factors for asthma (gender, a maternal and a paternal history of asthma), three asthma-associated features (eczema during preschool years, sensitisation by the age of 7 years, and susceptibility to respiratory infections during the first year of life), and six markers for single-nucleotide polymorphisms of the IL-4 receptor, were analysed in respect to these asthma phenotypes. 

Of the three inherited risk factors, the gender distribution was equal in the group with early onset asthma, whereas a female predominance was noticed in the late onset asthma group (Table 2). A maternal history of asthma was a risk factor for both asthma phenotypes, whereas a paternal history of asthma was only a risk factor for early onset asthma. The predominance of a paternal history of asthma in subjects with early onset asthma may, at least partly, be explained by the susceptibility to eczema associated with early onset asthma, as shown in Table 2. In contrast to a maternal history of asthma (OR 1.52, 95% CI 0.72–3.20), a paternal history of asthma was a risk factor for eczema in the offspring (OR 8.0, 95% CI 1.87–33.9). When adjusting for “a history of eczema during the child's first 7 years of life” in a multiple logistic regression model, the association expressed as OR between a paternal history of asthma and early onset asthma in the offspring was reduced, but not completely eliminated (OR 5.3, CI 1.16–24.6).

Sensitisation to inhaled allergens was common in both early onset and late onset asthma, that is, 2-3 times as common as in subjects without asthma (Table 2).

Of the six markers for single-nucleotide polymorphisms in the IL-4-receptor gene, three showed significant associations with the asthma phenotypes tested (Table 3). The AA genotype of the rs2057768 marker and the GG genotype of the rs1805010 marker were more commonly seen in late onset asthma, whereas the GG genotype of the rs2107356 marker was less common in early onset asthma.

## 4. Discussion

In this follow-up study of children from 7-8 years of age to young adulthood, we have subgrouped the subjects in the cohort into two asthma phenotype groups, separated by the age of onset of asthma, and one group without asthma. The two asthma phenotype groups were defined to be as similar as possible to the ones used in the Tucson cohort [8]. Even though there are similarities between the asthma phenotype groups, that is, they both represent persistent asthma and they are both associated with atopy, there were also phenotypic differences. Children with early onset asthma were more prone to recurrent infections and eczema during early childhood, as compared to children with late onset asthma, whereas the latter phenotypic group had a female predominance. We also found genotypic differences between the two phenotypes, except from the gender difference, in which the AA genotype of rs2057768 and the GG genotype of rs1805010 were more frequent in late onset asthma, whereas the GG genotype of rs2107356 was less common in early onset asthma.

These differences indicate that early onset asthma and late onset asthma, despite some phenotypic similarities, may be different endotypes and genotypes.

The susceptibility to develop early childhood eczema seems to be one pathological pathway separating the two phenotypes. This difference in eczema prevalence between the two subtypes not only was found in reported symptoms of eczema, but also was supported by hereditary differences. In this cohort, a paternal history of asthma increased the risk of eczema in the offspring, and we have previously shown that the AA genotype of rs2057768 is less common in children with eczema [9]. The susceptibility to respiratory infections during infancy may be a second endotypic or pathological difference between the phenotypes. This finding could be explained by the fact that children with early onset asthma already had developed asthma during the infancy period, and that common colds therefore cause more severe symptoms than in nonasthmatic children, symptoms that are easier to recall by the parents. But it may also be suggested that this tendency to develop early onset asthma, early eczema, and a concomitant susceptibility to respiratory infections during infancy, which are all manifestations or consequences of an immunological disturbance, is not seen to the same extent among children who later on develop late onset asthma. Such an explanation may be supported by the observation that infants with bronchiolitis tend to have a longer duration of disease, if they also have eczema [15], and a reduced interferon gamma production by antigen-stimulated cord blood mononuclear cells is seen in children who later on develop allergic disorders [16].

The pattern of risk factors found in our study is in concordance with the results from the original Tucson study by Martinez and coworkers [8], supporting the assumption that early onset asthma and late onset asthma might belong to different asthma phenotypes. As in the Tucson study, we found that both early and late onset asthma were associated with atopy and a history of maternal asthma, but only early onset asthma showed a close association with eczema. Even though the pattern of risk factors may indicate endotypic differences between the two phenotypes, the polymorphic differences found in the IL-4 receptor are a further support for such an assumption. We have previously reported, from the same cohort, that the AA genotype of rs2057768 was less common in children with asthma and concomitant eczema, as compared to those with an asthma phenotype not associated with eczema [9]. Since this finding seemed to be in contrast to results reported byHytönenand coworkers, who found that adult female patients with the A allele had more symptoms of active asthma [10], we concluded in our previous study that “severe atopic asthma in adult females may not necessarily be the same phenotype as childhood asthma with eczema” [9]. In this followup of the same cohort, with more specific asthma phenotypes defined, we can conclude that the assumption we then made seems to be correct; asthma with eczema in childhood is likely not the same phenotype as severe atopic asthma in adult females. Asthma with eczema in children is mainly seen in early onset asthma, whereas there is a clear female predominance in late onset asthma. Consequently, the A rs2057768 allele seems to indicate different biological consequences for different types of asthma. 

The markers rs2107356 and rs1805010 have previously been studied in adults with asthma, and the G alleles from these two markers were found to be more common in adult females with asthma [17]. In concordance with that study, we found a tendency for a G allele predominance in the female-dominated late onset asthma in our cohort. The significance of the low GG genotype frequency for rs2107356 seen in early onset asthma could, however, only be speculated on, since rs2107356, to our knowledge, has not been studied in relation to this specific asthma phenotype before.

Howthese genetic variants of the IL-4 receptor affect the disease characteristics of asthma is not known, but it might be linked to immune modulatory pathways. IL-4 is a proinflammatory cytokine stimulating IgE-antibody production and Th2-lymphocyte differentiation, and hence promoting asthma and allergy. These proinflammatory effects of IL-4 could, however, be downregulated by soluble IL-4 receptors, and soluble IL-4 receptors have been used to treat asthma [18, 19]. In one of the markers in our study (rs1805010), the GG genotype was in association with lower levels of soluble IL-4 receptors, as compared to both the AA and the AG genotypes (Hesselmar, unpublished data) possibly explaining why this genotype is more common among adult females with asthma [17] and late onset asthma.

Even though our findings support the accuracy of the Tucson-based classification system, separating persistent asthma into two different phenotypes depending on age of onset, our speculations on endotypic and genotypic differences between early onset and late onset asthma should be interpreted with caution. The analyses are based on a relatively small cohort of children and there is consequently quite a high risk of type II errors; true differences are not detected with statistical significance as the criterion. Additional differences between the groups, than those discussed, may exist. A particularly misleading reasoning appears if differences that come out as statistically significant are concluded to be larger than those that do not. This is an invalid interpretation. Furthermore, the retrospective collection of data covering the preschool period is a further limitation of the study giving opportunities for recall bias, and one may criticise the validity of the diagnosis of asthma since it was based on questionnaire replies only. However, we do believe that the questions about asthma used in the questionnaire have a high specificity for a diagnosis of asthma, since previous validation interviews at 12-13 years of age revealed that all children giving a positive answer to the question on current asthma used asthma medications, that is, inhaled *β*2-agonists and/or inhaled anti-inflammatory drugs [20]. We should also admit that due to expansion of known genetic risk factors in the development of asthma phenotypes, the heterogeneity of children's and adult asthma should be reexamined in well-constructed cohorts.

With these cautions in mind, our speculations about endotypic and genetic differences between the two asthma phenotypes should be considered as hypothesis generating, a hypothesis that needs to be tested in further studies.

## 5. Conclusions

We found that the asthma phenotypes “early onset asthma” and “late onset asthma” differ in several characteristics, suggesting that they may be of different endotypes and genotypes. Consequently, these two asthma phenotypes might be considered as two different disease entities in future studies on primary prevention of asthma and allergy.

## Figures and Tables

**Table 1:** Definitions and distributions of asthma phenotypes.

Phenotypes (*n* = 359)	Onset ≤3 Years	Onset >3 years	Asthma at 7 years	Asthma at 13 and/or 21 years
Healthy = no asthma (NA) (*n* = 318, 89%)	No	No	No	No
Early onset asthma (EOA) (*n* = 11, 3.1%)	Yes	—	—	Yes
Late onset asthma (LOA) (*n* = 30, 8.4%)	No	Yes	—	Yes

**Table 2:** Child-associated risk factors for asthma in relation to asthma phenotypes, as compared to subjects with no asthma.

Variable	NA^1^ *n* (%)	EOA^2^ *n* (%)	LOA^3^ *n* (%)
Gender			
Female	141 (44.3)	6 (54.5)	25 (83.3)
Male	177 (55.6)	5 (55.5)	5 (16.7)
*P* value^4^		0.549	<0.001
*P* value^5^	<0.001		

Mother with a history of asthma			
No	289 (93.0)	7 (70.0)	24 (82.8)
Yes	22 (7.0)	3 (30.0)	5 (17.2)
*P* value^4^		0.035	0.067
*P* value^5^	0.016		

Father with a history of asthma			
No	287 (94.7)	7 (70.0)	27 (96.4)
Yes	16 (5.3)	3 (30.0)	1 (3.6)
*P* value^4^		0.017	1.000
*P* value^5^	0.020		

A history of eczema during the first 7 years of age			
No	213 (68.1)	2 (18.2)	20 (66.7)
Yes	100 (31.9)	9 (81.8)	10 (33.3)
*P* value^4^		0.001	0.877
*P* value^5^	0.003		

Sensitisation by 7 years of age			
No	235 (77.8)	4 (36.4)	14 (53.8)
Yes	67 (22.2)	7 (63.6)	12 (46.2)
*P* value^4^		0.005	0.014
*P* value^5^	<0.001		

Number of colds during the first year of life			
≤5	265 (92.7)	5 (50.0)	21 (87.5)
>5	21 (7.3)	5 (50.0)	3 (12.5)
*P* value^4^		0.001	0.414
*P* value^5^	<0.001		

^1^No asthma, ^2^early onset asthma, ^3^late onset asthma.

^4^As compared to no asthma, *P* value based on chi-square, or Fisher's Exact Test.

^5^Between groups, *P* value based on Exact Test.

**Table 3:** IL-4 receptor polymorphisms and asthma phenotypes.

IL-4 receptor marker	NA^1^ *n* (%)	EOA^2^ *n* (%)	LOA^3^ *n* (%)
rs2057768			
AA	14 (7.2)	0 (0.0)	5 (33.3)
AG	74 (38.3)	2 (40.0)	5 (33.3)
GG	105 (54.5)	3 (60.0)	5 (33.3)
*P* value^4^		1.0	0.006
*P* value^5^	0.018		

rs2107356			
AA	38 (18.4)	1 (20.0)	1 (6.3)
AG	115 (55.6)	4 (80.0)	6 (37.5)
GG	54 (26.0)	0 (0.0)	9 (56.2)
*P* value^4^		0.001	0.414
*P* value^5^	<0.001		

rs3024632			
CC	0 (0.0)	0 (0.0)	0 (0.0)
CT	23 (11.2)	1 (20.0)	1 (6.7)
TT	182 (88.8)	4 (80.0)	14 (93.3)
*P* value^4^		1.000	0.711
*P* value^5^	0.897		

rs1805010			
AA	85 (38.1)	2 (33.3)	3 (18.7)
AG	117 (52.5)	4 (66.7)	6 (37.5)
GG	21 (9.4)	0 (0.0)	7 (43.8)
*P* value^4^		0.715	0.001
*P* value^5^	0.003		

rs1805015			
AA	157 (70.8)	3 (50.0)	16 (94.1)
AG	56 (25.2)	3 (50.0)	1 (5.9)
GG	9 (4.0)	0 (0.0)	0 (0.0)
*P* value^4^		0.487	0.093
*P* value^5^	0.138		

rs1801275			
CC	11 (4.9)	0 (0.0)	0 (0.0)
CT	78 (35.1)	3 (50.0)	5 (27.8)
TT	133 (60.0)	3 (50.0)	13 (72.2)
*P* value^4^		0.756	0.461
*P* value^5^	0.688		

^1^No asthma, ^2^early onset asthma, ^3^late onset asthma.

^4^As compared to no asthma, *P* value based on Monte Carlo Tests with 100000 iterations for 3 × 2 tables, and Exact Tests for 2 × 2 tables.

^5^Between all groups, *P* value based on Monte Carlo Test, with 100000 iterations.
